# Estimating Renal Function in the Elderly Malaysian Patients Attending Medical Outpatient Clinic: A Comparison between Creatinine Based and Cystatin-C Based Equations

**DOI:** 10.1155/2018/3081518

**Published:** 2018-05-13

**Authors:** Maisarah Jalalonmuhali, Salma Mohamed Abouzriba Elagel, Maw Pin Tan, Soo Kun Lim, Kok Peng Ng

**Affiliations:** ^1^Department of Medicine, University of Malaya Medical Centre, 59100 Kuala Lumpur, Malaysia; ^2^Management and Science University, 40100 Shah Alam, Selangor, Malaysia

## Abstract

**Background:**

To assess the performance of different GFR estimating equations, test the diagnostic value of serum cystatin-C, and compare the applicability of cystatin-C based equation with serum creatinine based equation for estimating GFR (eGFR) in comparison with measured GFR in the elderly Malaysian patients.

**Methods:**

A cross-sectional study recruiting volunteered patients 65 years and older attending medical outpatient clinic. 51 chromium EDTA (^51^Cr-EDTA) was used as measured GFR. The predictive capabilities of Cockcroft-Gault equation corrected for body surface area (CGBSA), four-variable Modification of Diet in Renal Disease (4-MDRD), and Chronic Kidney Disease Epidemiology Collaboration (CKD-EPI) equations using serum creatinine (CKD-EPIcr) as well as serum cystatin-C (CKD-EPIcys) were calculated.

**Results:**

A total of 40 patients, 77.5% male, with mean measured GFR 41.2 ± 18.9 ml/min/1.73 m^2^ were enrolled. Mean bias was the smallest for 4-MDRD; meanwhile, CKD-EPIcr had the highest precision and accuracy with lower limit of agreement among other equations. CKD-EPIcys equation did not show any improvement in GFR estimation in comparison to CKD-EPIcr and MDRD.

**Conclusion:**

The CKD-EPIcr formula appears to be more accurate and correlates better with measured GFR in this cohort of elderly patients.

## 1. Background

Prevalence of chronic kidney disease (CKD) in the elderly is increasing globally as life expectancy continues to improve [1]. According to the latest report from the Department of Statistics Malaysia, life expectancy at birth in Malaysia is 72.7 years for male and 77.4 years for female [2]. From previous literatures, we know that serum creatinine alone is imprecise to assess kidney function in the elderly [3]. Apart from age, the level can also be affected by gender, muscle mass, diet, and tubular creatinine secretion particularly at reduced glomerular filtration rate (GFR); this is especially important in older people. Serum creatinine based equation such as Cockcroft-Gault corrected for body surface area (CGBSA), four-variable Modification of Diet in Renal Disease (4-MDRD), and Chronic Kidney Disease Epidemiology Collaboration (CKD-EPI) equation is widely used to overcome the shortcomings of serum creatinine alone in estimating GFR [4–6].

In this part of the world, creatinine based formula remained an important tool for assessment of kidney function. The main bulk of patients are being treated at the peripheral hospitals and clinics by the primary healthcare practitioner. A small proportion will present to major hospitals during illnesses and later transferred over back to the community for further management. This is partly due to the difficulty transporting elderly patients to major hospitals which are usually situated in the cities. Therefore, the use of GFR estimating equations in the out-patient settings will help the healthcare practitioner in deciding the need and timing of referrals for subspecialty management.

Serum cystatin-C is a small molecular weight protein produced by all nucleated cells, freely filtered by glomerulus then reabsorbed and completely degraded (but not secreted) by proximal tubules [7, 8]. It is present in serum, saliva, semen, urine, and cerebrospinal fluid and the level is not affected by muscle mass, and hence it is ideal to be used in elderly patients especially those with malnutrition. In a meta-analysis by Dharnidharka et al., serum cystatin-C was found to be superior to serum creatinine as a marker of GFR [9]. The use of cystatin-C in estimating GFR in this country is still very limited mainly due to its availability. It is interesting to look at the performance of different creatinine based equations against cystatin-C based equation in estimating GFR in our multiethnic elderly cohort.

## 2. Methods

This is a cross-sectional study recruiting elderly patients aged 65 years and older seen in University of Malaya Medical Centre (UMMC) medical outpatient clinic. Participation in this study is on voluntary basis and a written informed consent was taken from each participant. Patients were excluded if they have any of the following: (a) acute kidney injury, (b) inability to consent, (c) history of limb amputation, (d) physical disability rendering weight and height measurement difficult, (e) oedema, fluid overload, and nephrotic syndrome, and (f) physical condition that renders phlebotomy for blood samples and/or peripheral line insertion difficult. The study was approved by UMMC Medical Ethics Committee (UMEC) in accordance with the Helsinki Declaration under MECID number 20145-324.

### 2.1. GFR Measurement

GFR was determined using plasma clearance of 51 chromium ethylenediamine tetraacetic acid (^51^Cr-EDTA), which was injected as a single bolus intravenously into patients. Four blood samples were taken at 2 hours, 2.5 hours, 3 hours, and 4 hours after ^51^Cr-EDTA injection from the opposite upper limb. Patient's height and weight were measured for body surface area (BSA) calculation. GFR was calculated using the slope-intercept method and normalized to BSA, which was calculated using du Bois formula. The result was then corrected using Brochner-Mortensen equation.

Volume distribution (Vd) is calculated by

(1) Vd=Standard  activitycpm×weight  of  dose×100 mlPocpm×weight  of  standard

- Standard activity is calculated using computer generated chromium result.
- Weight of dose is calculated from weight of syringe and dose before injection – after injection.
- Po (zero time plasma activity) is corrected by extrapolating the curve to zero time.

Slope clearance (C-slope) is calculated by

(2) $$\begin{array}{c} \frac{\text{C-slope}}{\text{slope intercept}}=\frac{0.693}{T^{1/2}}\times Vd \end{array}$$

Normalized GFR is calculated by

(3) $$\begin{array}{c} \text{Normalized GFR}=\frac{\text{C-slope}}{\text{Patient's BSA}}\times 1.73 \end{array}$$

### 2.2. GFR Estimation

Table 1 showed the different equations used for comparison in this study. All patients had their serum creatinine and serum cystatin-C blood test withdrawn during the peripheral venous access insertion. Measurement of serum creatinine was performed using enzymatic creatinine assay based on the enzymatic reaction [10] with normal adult reference range of 53–97 *μ*mol/L for male and 44–71 *μ*mol/L for female. The creatinine values were adjusted to isotope dilution mass spectrometry (IDMS) traceable assay. Measurement of serum cystatin-C was performed at an independent pathology laboratory outside the hospital using an automated particle-enhanced immune-nephelometry method with normal reference range of 0.85 mg/l or less using N Latex Cystatin-C from Siemens Healthcare Diagnostics Products GmbH (Germany). N Latex Cystatin-C values were then converted to eGFR based on the Hoek equation. The adjusted Hoek equation to calculate the eGFR is GFR (ml/min/1.73 m^2^) = −4.32 + 80.35/cys-C [11].

### 2.3. Statistical Analysis

SPSS version 20.0 was used to calculate baseline characteristics frequency, mean, median, range, and standard deviation. Mean GFR were given with a 95% confidence interval (CI) unless indicated otherwise. *P* values < 0.05 were considered significant. Pearson's correlation coefficients (*r*) were calculated between ^51^Cr-EDTA clearance and estimated GFR by a linear correlation analysis. Pairwise comparison of the mean was performed using paired *t*-test.

Bias, precision, and accuracy within 30% of the measured GFR were determined. Bias is defined as mean difference between estimated GFR and the measured GFR (^51^Cr-EDTA). The precision of the estimates was determined as SD of the mean difference between measured GFR and eGFR. Accuracy was determined by integrating precision and bias and was calculated as the percentage of GFR estimates within 30% of the measured GFR. Moreover, a graphical analysis was carried out using Bland and Altman plots. This was used to assess the limits of agreement between the eGFR and the measured GFR.

Accuracy is the most important determinants for a good estimated GFR and best supported by lower bias, greater precision, and lower limits of agreement. However, as we understand that bias, precision and limits of agreement may be affected by the overall means and outliers; therefore, the individual parameter may not reflect the best estimated GFR.

## 3. Results

A total of 40 elderly patients with mean age of 73.1 ± 5.9 years and predominantly male 31 (77.5%) were recruited. Mean serum creatinine was 165.7 ± 60 *μ*mol/L, cystatin-C was 1.6 ± 0.5 mg/l, and mean measured GFR was 41.2 ± 18.9 ml/min/1.73 m^2^. 34 out of 40 patients (85%) had measured GFR < 60 ml/min/1.73 m^2^ with mean of 34.97 ± 10.2 ml/min/1.73 m^2^ with 13 (32.5%) of them with measured GFR of <30 ml/min/1.73 m^2^. Baseline demographics of study population are shown in Table 2.

All the eGFR equations, namely, CGBSA, 4-MDRD, CKD-EPIcr, and CKD-EPIcys, correlated well with measured GFR (^51^Cr-EDTA). The 4-MDRD equation had the lowest bias followed by CKD-EPIcr, CKD-EPIcys, and CGBSA with bias of −2.76, −3.09, 4.51, and −7.97 ml/min/1.73 m^2^, respectively. In this study we found that CKD-EPIcr seems to be more precise (8.18 ml/min/1.73 m^2^) followed by CKD-EPIcys (8.26 ml/min/1.73 m^2^), 4-MDRD (8.70 ml/min/1.73 m^2^), and CGBSA (10.68 ml/min/1.73 m^2^). CKD-EPIcr was found to have the higher accuracy as compared to the others. The performances of the estimated GFR equations are tabulated in Table 3 for reference.

**Figure 1:**
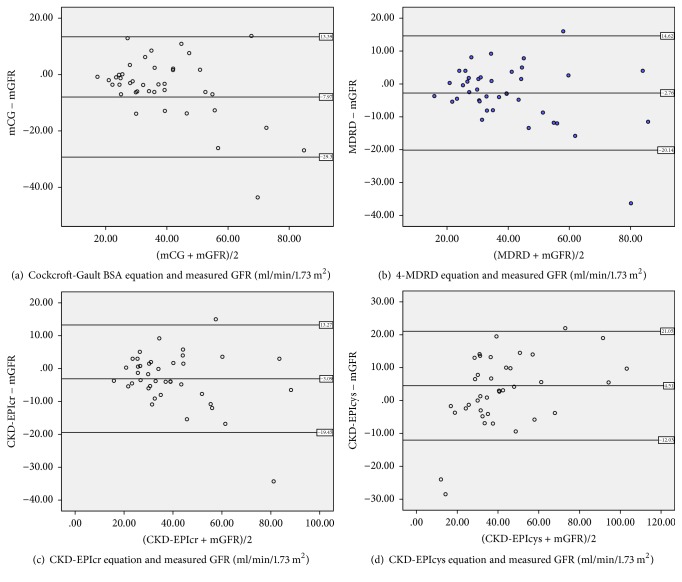
(a–d) Bland and Altman analysis of GFR estimates. In this analysis, the differences between estimated and measured GFR are plotted against the average of the estimated and measured GFR for each individual patient.

The differences between estimated and measured GFR were illustrated using a graphical technique according to Bland and Altman plot (Figures 1(a)–1(d)). These figures display the span between +2SD and −2SD of the mean difference (limits of agreement between 2 methods), which represent 95% CI. Smaller limits of agreement were found for the CKD-EPIcr (32.67 ml/min/1.73 m^2^), followed by CKD-EPIcys (33.06 ml/min/1.73 m^2^), 4-MDRD (34.76 ml/min/1.73 m^2^), and CGBSA (42.69 ml/min/1.73 m^2^) equation. Limits of agreement can also be affected by the extreme outliers. Therefore, a sum of all the parameters has to be considered to determine the more accurate eGFR equation in comparison with ^51^Cr-EDTA in our study cohort.

Overall, we can conclude that CGBSA, 4-MDRD, CKD-EPIcr, and CKD-EPIcys have an excellent correlation with measured GFR and it can be used in our daily clinical practice. Further analysis revealed that CKD-EPIcr seems to perform better as compared to other equations in the elderly populations with reasonably low bias, greater precision, and accuracy. It is further supported by lower limits of agreement and less scattering of the Bland-Altman plot. CKD-EPIcys equation unexpectedly did not perform very well in our study probably because of the different standardization of the cystatin-C value used in the original CKD-EPIcys equation.

## 4. Discussion

It has been shown that GFR decreases with aging, age-related changes in the renal function, and progressive loss of muscle mass with aging (sarcopenia) [12, 13]. Accurate measurement of renal function is mandatory for appropriate drug dosing and contrast related procedure. Apart from that, it is a well-known fact that CKD in the elderly populations is associated with frailty and poor physical performance [14]. Frailty and its progression have a significant impact on the mortality among the elderly [15]. Hence, an accurate GFR measurement is an important clinical assessment in daily clinical practice particularly in this cohort.

Direct GFR measurement is the gold standard of kidney function assessment. However, it is costly and time consuming and the need for multiple bloods sampling makes it impractical. Since GFR estimation in elderly is a topic of ongoing debate, we conducted this study to evaluate the performance of different GFR estimating equations in comparison with gold standard radio-labelled measurement of ^51^Cr-EDTA clearance in our multiethnic cohort of elderly patients. We have also included Cockroft-Gault equation into this study because it is still commonly used by many even until now. One must remember that Cockcroft-Gault equation intends to measure the creatinine clearance. In contrast, other eGFR equations are measuring the GFR instead. Tubular secretion, extrarenal clearance of creatinine, and drugs affecting the renal handling of creatinine will result in inaccurate creatinine clearance. That is why creatinine clearance always gives higher values than GFR, while Cockcroft-Gault equation provides lower values than GFR equations. Age and weight are the main reasons of discrepancy. Therefore, Cockcroft-Gault and other eGFR equations cannot be used interchangeably to estimate kidney function [16].

The findings from this study are consistent with the work done by Stevens et al., which showed that CKD-EPIcr was better than MDRD even in estimating GFR > 60 ml/min/1.73 m^2^ [17]. Particularly in subgroup of >65 years old, CKD-EPIcr has lower bias throughout all CKD stages. We reported that CKD-EPIcr is more accurate with reasonably low bias, greater precision, accuracy, and lower limits of agreement as compared with the measured GFR.

It is unlikely that one single eGFR equation will work equally well in different cohort of patients. In particular, the use of eGFR equations in the elderly is more challenging as it can be affected by physiological change of aging process. It is worth to note that both the MDRD and CKD-EPI study were performed in a much younger population. The mean age of patients in MDRD and CKD-EPI study was 52 and 47 years, respectively [6, 18]. The elderly were underrepresented in both studies, hence leaving a gap in the evidence.

We have also explored the practicality of using cystatin-C as an endogenous marker alternative to serum creatinine. It is mainly filtered by the kidney and achieved 94% of renal clearance calculated using ^51^Cr-EDTA in previous literatures [9, 19–21]. Although it can be affected by renal tubular catabolism, reabsorption, use of systemic glucocorticoids, and thyroid dysfunction, cystatin-C was proposed to be better in comparison to serum creatinine as an endogenous marker of GFR [9].

Comparison between creatinine based and cystatin-C based equation using iohexol as exogenous marker of GFR in elderly patients was studied by Kilbride et al. [22]. In a cohort of 394 elderly patients with median age of 80 years, CKD-EPIcr equation was less biased and more accurate than the MDRD. None of the equations including CKD-EPI using cystatin-C alone or in combination with creatinine concentration achieved an ideal accuracy within 30% in the overall population of that study. Similarly, this present study also failed to document the advantage of using cystatin-C based equation over creatinine based equation in estimating GFR in the elderly. By right, incorporation of cystatin-C in eGFR equations is supposed to have better performance due to its unique properties as outlined before. Nevertheless, our study population was too small to draw any strong conclusion with regard to the utility of cystatin-C based equation.

## 5. Limitations of the Study

The authors would like to emphasize that this is a small uni-centre cohort of CKD patients, predominantly male and mainly consisted of CKD stages 3 and 4. A balanced number of patients in different GFR groups could not be controlled due to the continuous sampling method used in this study. We recommend a larger and more robust study to ascertain the validity of this study results specifically in our multiethnic cohort of South East Asian population. Although this study has the above-mentioned limitations, this is the first study to be conducted in Malaysia using ^51^Cr-EDTA as reference GFR and cystatin-C among the elderly patients.

## 6. Conclusion

We conclude that CKD-EPIcr formula appeared to be more accurate and correlates better with measured GFR in this cohort of Malaysian elderly. The expected advantage of cystatin-C based equation was not observed in this present study possibly because of the limitations stated before. The use of commonly available eGFR equations, namely, 4-MDRD and CKD-EPIcr, should be encouraged for better kidney function assessment. Cockcroft-Gault equation in the elderly leads to overestimation of GFR and may pose significant adverse situations. Further studies should be done to ascertain the best GFR estimation formula in our multiethnic elderly population especially looking at the potential role of cystatin-C for kidney function assessment.

## Figures and Tables

**Table 1:** Different eGFR formula according to gender.

eGFR methods	Gender	Equations
Cockcroft-Gault BSA (CGBSA)	Male Female	$\left.\begin{array}{c} \left((140-\text{Age})\times \text{mass}\left(kg\right)\times 1.23\right)/\text{Serum Creatinine}(µmol/L)\\ \left((140-\text{Age})\times \text{mass}\left(kg\right)\times 1.04\right)/\text{Serum Creatinine}(µmol/L) \end{array}\right\}\times 1.73/BSA$

4-MDRD	Male	175 × (Serum Creatinine/88.4)^−1.154^ × Age^−0.203^ × {1.212 if Black}
	Female	175 × (Serum Creatinine/88.4)^−1.154^ × Age^−0.203^ × {1.212 if Black} × 0.742
		(Serum Creatinine in *µ*mol/L)

CKD-EPIcr (creatinine)	Male	141 × min(SCr/0.9,1)^−0.411^ × max(SCr/0.9,1)^−1.209^ × 0.993^Age^ × {1.159 if Black}
	Female	141 × min(SCr/0.7,1)^−0.329^ × max(SCr/0.7,1)^−1.209^ × 0.993^Age^ × {1.159 if Black} × 1.018

CKD-EPIcys (cystatin-c)	Male	133 × min(Scys/0.8,1)^−0.499^ × max(Scys/0.8,1)^−1.328^ × 0.996^Age^
	Female	133 × min(Scys/0.8,1)^−0.499^ × max(Scys/0.8,1)^−1.328^ × 0.996^Age^ × 0.932

**Table 2:** Patient's baseline characteristics.

Characteristic (*n* = 40)	Mean + SD (median) or *n* (%)
Male	31 (77.5%)
Age (year)	73.1 ± 5.9
Age category	
<75	28 (70.0%)
≥75	12 (30.0%)
Weight (kg)	69.16 ± 13.32
BMI (kg/m^2^)	26.5 ± 4.6
BSA (m^2^)	1.70 ± 0.2
Serum creatinine (umol/l)	165.7 ± 60.0
Serum cystatin-C (mg/l)	1.6 ± 0.5
Measured GFR (ml/min/1.73 m^2^)	41.2 ± 18.9
CKD staging (ml/min/1.73 m^2^)	
<60	34 (85.0%)
≥60	6 (15.0%)
Medical history	
Diabetes mellitus	17 (42.5%)
Hypertension	28 (70.0%)

**Table 3:** Correlation coefficient (*r*), mean, bias, precision, and accuracy for CGBSA, 4-MDRD, CKD-EPIcr, and CKD-EPIcys equations.

	Correlation coefficient (*r*)	Mean GFR (ml/min/1.73 m^2^)	Range (IQR)		Mean difference (bias)	SD of mean bias (precision)	Accuracy within 30%
			Lower	Upper			
Measured GFR		41.2	17.7	98.3			
CGBSA	0.861^*∗*^	33.2	15.4	65.6	−7.97	10.68	85
4-MDRD	0.889^*∗*^	38.4	14.0	86.0	−2.76	8.70	90
CKD-EPIcr	0.902^*∗*^	38.1	14.0	85.0	−3.09	8.18	95
CKD-EPIcys	0.928^*∗*^	46.1	16.0	108.0	4.90	8.26	81

^*∗*^Significantly correlates with *P* < 0.001 (bias: mean difference of estimated GFR and measured GFR; accuracy: *n* percentage of GFR estimates within *n*% of measured GFR; IQR: interquartile range).

## Abbreviations

CKD: Chronic kidney disease
GFR: Glomerular filtration rate
CGBSA: Cockcroft-Gault corrected for body surface area
4-MDRD: Modification of diet in renal disease
CKD-EPI: Chronic Kidney Disease Epidemiology Collaboration
^51^Cr EDTA: 51-chromium ethylenediamine tetraacetic acid
mGFR: Measured glomerular filtration rate
BSA: Body surface area
CKD-EPIcr: CKD-EPI creatinine
CKD-EPIcys: CKD-EPI cystatin.

## References

[1] Mallappallil M., Friedman E. A., Delano B. G., McFarlane S. I., Salifu M. O. (2014). Chronic kidney disease in the elderly: evaluation and management.

[2] https://www.dosm.gov.my

[3] Swedko P. J., Clark H. D., Paramsothy K., Akbari A. (2003). Serum creatinine is an inadequate screening test for renal failure in elderly patients.

[4] Levey A. S., Bosch J. P., Lewis J. B., Greene T., Rogers N., Roth D. (1999). A more accurate method to estimate glomerular filtration rate from serum creatinine: a new prediction equation. Modification of Diet in Renal Disease Study Group.

[5] Poggio E. D., Wang X., Greene T., Van Lente F., Hall P. M. (2005). Performance of the modification of diet in renal disease and Cockcroft-Gault equations in the estimation of GFR in health and in chronic kidney disease.

[6] Levey A. S., Stevens L. A., Schmid C. H. (2009). A new equation to estimate glomerular filtration rate.

[7] Randers E., Kristensen J. H., Erlandsen E. J., Danielsen H. (1998). Serum cystatin C as a marker of the renal function.

[8] Westhuyzen J. (2006). Cystatin C: a promising marker and predictor of impaired renal function.

[9] Dharnidharka V. R., Kwon C., Stevens G. (2002). Serum cystatin C is superior to serum creatinine as a marker of kidney function: a meta-analysis.

[10] Fossati P., Prencipe L., Berti G. (1983). Enzymic creatinine assay: A new colorimetric method based on hydrogen peroxide measurement.

[11] Hoek F. J., Kemperman F. A. W., Krediet R. T. (2003). A comparison between cystatin C, plasma creatinine and the Cockcroft and Gault formula for the estimation of glomerular filtration rate.

[12] Van Pottelbergh G., Vaes B., Morelle J., Jadoul M., Wallemacq P., Degryse J. (2011). Estimating GFR in the oldest old: does it matter what equation we use?.

[13] Van Pottelbergh G., Van Heden L., Matheï C., Degryse J. (2010). Methods to evaluate renal function in elderly patients: a systematic literature review.

[14] Reese P. P., Cappola A. R., Shults J. (2013). Physical performance and frailty in chronic kidney disease.

[15] Buchman A. S., Wilson R. S., Bienias J. L., Bennett D. A. (2009). Change in frailty and risk of death in older persons.

[16] Pedone C., Corsonello A., Incalzi R. A. (2006). Estimating renal function in older people: A comparison of three formulas.

[17] Stevens L. A., Schmid C. H., Greene T. (2010). Comparative performance of the CKD Epidemiology Collaboration (CKD-EPI) and the Modification of Diet in Renal Disease (MDRD) Study equations for estimating GFR levels above 60 mL/min/1.73 m2.

[18] Klahr S., Levey A. S., Beck G. J. (1994). The effects of dietary protein restriction and blood-pressure control on the progression of chronic renal disease.

[19] Grubb A. O. (2000). Cystatin C—properties and use as diagnostic marker.

[20] Tenstad O., Roald A. B., Grubb A., Aukland K. (1996). Renal handling of radiolabelled human cystatin C in the rat.

[21] Jacobsson B., Lignelid H., Bergerheim U. S. R. (1995). Transthyretin and cystatin C are catabolized in proximal tubular epithelial cells and the proteins are not useful as markers for renal cell carcinomas.

[22] Kilbride H. S., Stevens P. E., Eaglestone G. (2013). Accuracy of the MDRD (Modification of Diet in Renal Disease) study and CKD-EPI (CKD Epidemiology Collaboration) equations for estimation of GFR in the elderly.

